# Relationship between Postoperative Complications and Ratio of Amount of Wetting Solution to Ideal Body Weight in Liposuction Procedures

**DOI:** 10.3390/jpm14050494

**Published:** 2024-05-07

**Authors:** Serap Aktas Yildirim, Lerzan Dogan, Zeynep Tugce Sarikaya, Bulent Gucyetmez, Yener Demirtas, Fevzi Toraman

**Affiliations:** 1 Department of Anesthesiology and Reanimation, Acibadem Mehmet Ali Aydinlar University School of Medicine, Istanbul 34752, Turkey; 2 Department of Anesthesiology and Reanimation, Acibadem Altunizade Hospital, Istanbul 34662, Turkey; 3 Private Practice, Atasehir, Istanbul 34746, Turkey

**Keywords:** high-volume liposuction, ideal body weight, postoperative complications

## Abstract

Background: The use of wetting solutions (WSs) during high-volume liposuction is standard; however, the optimal amount of WS and its components and their effect on postoperative complications are unclear. We evaluated the effect of a WS and its components, calculated according to ideal body weight (IBW), on postoperative complications. Methods: High-volume liposuction with a WS containing 0.5 g of lidocaine and 0.5 mg of epinephrine in each liter was performed in 192 patients. Patients who received ≤90 mL/kg of WS were designated as group I and those who received >90 mL/kg of WS as group II. Postoperative complications and adverse events that occurred until discharge were recorded. Results: The mean total amount of epinephrine in the WS was significantly higher for group II (3.5 mg; range, 3.0–4.0 mg) than for group I (2.0 mg; range, 1.8–2.5 mg; *p* < 0.001), as was the mean total amount of lidocaine (3.5 g [range, 3.5–4.3 g] vs. 2.0 g [range, 1.8–2.5 g], respectively; *p* < 0.001). No major cardiac or pulmonary complications occurred in either group. Administration of >90 mL/kg of WS increased the median risk of postoperative nausea 5.3-fold (range, 1.8- to 15.6-fold), that of hypertension 4.9-fold (range, 1.1- to 17.7-fold), and that of hypothermia 4.2-fold (range, 1.1- to 18.5-fold). The two groups had similar postoperative pain scores and blood transfusion rates. Conclusions: The risks of postoperative nausea, vomiting, hypothermia, and hypertension may increase in patients who receive >90 mL/kg of WS calculated according to IBW during high-volume liposuction.

## 1. Introduction

In liposuction, one of the most popular cosmetic surgical procedures worldwide, a significant amount of adipose tissue can be removed. Liposuction is generally viewed as a benign procedure, but it can be considered major surgery because of risk factors such as long operation times, intraoperative volume shifts, hypothermia, the use of high doses of epinephrine and lidocaine in the wetting solution (WS), and the possibility of fatal cardiac or pulmonary complications [1]. Therefore, anesthesiologists and plastic surgeons should be aware of the intraoperative and postoperative pathophysiological changes caused by liposuction. In the literature, large-volume liposuction is defined as the removal of more than 5 L of lipoaspirate in a single procedure [2]. The most suitable candidates for liposuction are individuals whose body mass index (BMI) is <30 kg/m^2^, but large-volume liposuction is applied more frequently to individuals who are overweight and obese. However, obesity can cause pathophysiological changes. In particular, increased cardiac output and changes in the distribution volume may affect the pharmacokinetic and pharmacodynamic behavior of drugs [3]. Liposuction-related perioperative complication rates may also be high, particularly in individuals with obesity [4]. Medications administered according to the actual body weight (ABW) may lead to adverse outcomes of overdose. Therefore, some authors recommend adjusting perioperative medications according to the ideal body weight (IBW) instead of ABW [5]. The effects of WS and its components used in liposuction on patient outcomes have been examined in many studies but the IBW of patients has not been considered [6,7]. We hypothesized that during high-volume liposuction, standardized WS and its components applied regardless of the ideal body weight of the patients may increase postoperative complications. This study retrospectively analyzed patients who underwent high-volume liposuction with a standardized WS, calculated the WS used in these patients according to ideal body weight, and examined its effect on early postoperative complications and adverse events.

## 2. Materials and Methods

### 2.1. Ethical Approval

This retrospective trial was conducted between September 2021 and September 2023 at Acibadem Altunizade Hospital, which belongs to Acibadem MAA University, Istanbul, Turkey. Ethical approval was obtained from the regional ethics committee of Acibadem MAA University (ATADEK-2023-13/463) on 17 August 2023. This study complied with the ethical principles for medical research involving human subjects according to the Declaration of Helsinki. All patients provided informed consent before the data were collected.

### 2.2. Trial Registration

The trial was registered (No. NCT06260501) on 14 February 2024.

### 2.3. Patients

Patients who underwent liposuction with more than 5 L of adipose tissue aspiration between 2021 and 2023 under general anesthesia were included in the study. Patients younger than 18 years were excluded from the study. Patients were divided into two groups according to the IBW-based amounts of WS administered: those who received ≤90 mL/kg of WS (group I) and those who received >90 mL/kg of WS (group II). IBW was calculated according to the Devine equation (kg): 45.5 + (0.91 × [height (cm) × 152.4]) [8].

We examined each patient’s digital records of their hospitalizations and recorded their ages, heights, weights, BMI, comorbidities, preoperative hemoglobin levels, and medications. We also recorded the amount of intravenous fluid administered intraoperatively, the urine output, the duration of surgery, the amount of adipose tissue aspirated, the amount of epinephrine and local anesthetic in the liposuction solution, the body temperature at the end of surgery, and time until discharge from the recovery room, as well as intraoperative adverse events such as hypoxia and hypotension; postoperative adverse events such as nausea, vomiting, hypotension, hypoxia, pulmonary embolism, myocardial infarction, and arrhythmia; blood transfusions; postoperative pain; and length of hospitalization. The Numerical Pain Rating Scale was used to assess postoperative pain levels [9]. Mean arterial pressure (MAP) above 105 mmHg was considered hypertension, and a value below 65 mmHg was considered hypotension [10,11]. In the postoperative period, patients with hemoglobin (Hb) levels below 8 g/dL and patients with Hb levels between 8 and 10 g/dL with symptoms such as tachycardia and hypotension received blood transfusions.

### 2.4. Anesthesia

General anesthesia was induced in all patients, and blood pressure, electrocardiograms, blood oxygen saturation, body temperature, and urine output during surgery were continuously monitored non-invasively. Anesthesia was maintained with a mixture of 40% oxygen, 60% room air, and sevoflurane with a minimum alveolar concentration of 0.9–1. Remifentanil was infused for intraoperative analgesia in the range of 0.02–0.5 μg/kg/min. To maintain anesthesia depth between Bispectral indexes of 40 and 60, a BIS Vista Monitor (Medtronic, Swedesboro, NJ, USA) was used for monitoring consciousness. Ringer’s lactate solution was administered as an intravenous fluid, and its rate and amount were adjusted according to hemodynamic findings. All patients received ondansetron, 8 mg, intravenously 30 min before the end of surgery. Intravenous fluids were warmed for all patients, and areas outside the surgical field were heated to a temperature of 43 °C by a forced-air warming blanket (Bair Hugger^®^; 3M, St. Paul, MN, USA). At the end of surgery, the patients were extubated and transferred to the post-anesthesia care unit (PACU). All patients were transferred from the PACU to their rooms after they were hemodynamically stable and normothermia was achieved.

### 2.5. Surgical Technique

After anesthesia induction, the patients were placed either in a prone position for the first part of the procedure and then in a supine position for the second part or only in a supine position, depending on the liposuction area. The super-wet technique (aspiration of 1 mL of fat per 1 mL of infiltrate) was used during liposuction in all patients. As a WS, Ringer’s lactate solution was used with 500 mg of lidocaine and 0.5 mg of epinephrine in each liter. Operative room temperature (22 °C) wetting fluid was instilled into the superficial and deep layers, and ultrasound-assisted liposuction (UAL) with a vibration amplification of sound energy at resonance (VASER) liposuction system (Solta Medical, Bothell, DC, USA) was performed on all targeted body areas of the patients in both groups. Multi-hole blunt-tipped fat aspiration cannulas with 3.7–4.6 mm in diameter and 30 cm in length were used. Liposuction was performed on 5 different areas: abdomen, hips, back, thighs, and arms. All liposuction procedures were performed by the same experienced surgeon.

### 2.6. Statistical Analysis

Data were calculated as means with standard deviations, medians with ranges (representing quartiles), and percentages. Student’s *t*-test, the Mann–Whitney *U* test, and the chi-square test were used for group comparisons. The estimated power was calculated as 0.80 for the percentages of postoperative MAP >105 mmHg through an independent proportions test (sample size for group I was 54 and that for group II was 138; a postoperative MAP >105 mmHg had to be achieved in 3.7% of group I and 18.1% of group II). To calculate the relative risks for postoperative nausea, hypertension, and hypothermia as a result of an IBW-based WS of >90 mL/kg, we used the *Z* test. IBM SPSS Statistics for Windows, Version 29 (Armonk, NY, USA; released 2022), was used for statistical analysis. Significance was represented by *p*-values <0.05.

## 3. Results

We retrospectively examined the data of 192 patients in whom at least 5 L of adipose tissue was aspirated during liposuction. The first quartile of IBW-based WS was 0–90 mL/kg. The patients were divided into two groups: those in whom the amount of IBW-based WS was ≤90 mL/kg (WS/IBW ≤ 90 mL/kg; group I) and those in whom the amount of WS was >90 mL/kg (WS/IBW > 90 mL/kg; group II).

All patients were female, their median age was 39 years (range, 31–47 years), and their median BMI was 29.5 kg/m^2^ (range, 25.4–34.6 kg/m^2^). The BMI of 39 patients (20.3%) was <25 kg/m^2^, and that of 153 (79.7%) exceeded 25 kg/m^2^. All patients had an American Society of Anesthesiologists score <2, and 20.5% had a history of hypothyroidism. Approximately 70% of the patients had no comorbidities. The average surgical time was 230 min (range, 200–265 min), and the average amount of lipoaspirate was 7.3 L (range, 6.0–8.5 L). The average amount of fluid administered during surgery was 1850 mL (range, 1500–2000 mL), and the average amount of WS administered was 6.3 mL (range, 5.0–7.5 mL). No major pulmonary complications (e.g., embolism and pneumonia) or cardiac complications (e.g., myocardial infarction and arrhythmia) occurred. All patients were hypothermic at the end of surgery (body temperature < 35.3 °C). The most common postoperative complication was nausea (23.4%). Twenty percent of patients scored pain above 5 on the Numerical Pain Rating Scale after surgery. Twenty-five patients (13%) received a blood transfusion during the postoperative period. Patient demographic data, preoperative and postoperative hemoglobin levels, amount of WS and components administered, duration of stay in the PACU, and postoperative complications are listed in Table 1.

In comparing patients with regard to the amount of WS administered according to IBW, we found that groups I and II had similar demographic data (except BMI), American Society of Anesthesiologists scores, comorbidities, hemoglobin levels, and lengths of hospitalization. The median BMI of patients in group II (31.6 kg/m^2^; range, 27.3–35.5 kg/m^2^) was higher than that in group I (25.1 kg/m^2^; range, 23.6–28.6 kg/m^2^; *p* < 0.01). The IBW values of the patients in both groups were similar. BMI >30 kg/m^2^ characterized 20.4% of patients in group I, in contrast to 58.7% of those in group II. The median amount of lipoaspirate was higher in group II (8.0 L; range, 7.0–9.0 L) than in group I (5.0 L; range, 4.5–6.0 L; *p* < 0.001). The mean total amount of epinephrine in the WS was also significantly higher in group II (3.5 mg [corresponding to 0.043 mg/kg for ABW]; range, 3.0–4.0 mg) than in group I (2.0 mg [corresponding to 0.031 mg/kg for ABW]; range, 1.8–2.5 mg; *p* < 0.001), as was the mean total amount of lidocaine in the WS (in group II, 3.5 g range, 3.5–4.3 g; in group I, 2.0 g; range, 1.8–2.5 g; *p* < 0.001). When calculated according to both IBW and ABW, the total amounts of epinephrine and lidocaine administered were significantly higher in group II than in group I (*p* < 0.001). The total amounts of epinephrine and lidocaine in the WS and the comparison of the amounts calculated according to IBW and ABW between the groups are presented in Table 2.

Regarding the site of liposuction, 84.8% of patients in group II underwent liposuction in three or more areas, while 83.3% of patients in group I underwent liposuction in two areas. While all patients in group II were bilateral cases, all except five patients in group I were bilateral cases. The surgery was significantly longer for group II than for group I (*p* < 0.001), as was the stay in the PACU (*p* < 0.0006). At the end of surgery, the body temperature of the patients in group II was lower than in group I (*p* < 0.002). Group II exhibited more postoperative nausea (*p* < 0.001), vomiting (*p* = 0.041), and hypertension (MAP > 105 mm/Hg; *p* = 0.021) than group I. In both groups, in patients who developed postoperative hypertension, MAP decreased below 105 mm Hg in the first six hours postoperatively without requiring any treatment. Postoperative pain scores and blood transfusion rates were similar for the two groups. In the patients in group II, the median risk of postoperative nausea was 5.3-fold higher than for those in group I (range, 1.8–15.6; *p* = 0.003), the risk of hypertension was 4.9-fold higher (range, 1.2–19.8; *p* = 0.027), and the risk of hypothermia was 4.2-fold higher (range, 1.1–18.5; *p* = 0.045). The demographic data of the patients in groups I and II and the comparison of these data are presented in Table 2. The relative risks of nausea, hypothermia, and hypertension in patients who received >90 mL/kg of WS are listed in Table 3.

## 4. Discussion

In this study, stays in the PACU were longer, and postoperative adverse outcomes such as hypertension, nausea, vomiting, and hypothermia were more common among patients who received >90 mL/kg of WS (group II) during liposuction than among those who received ≤90 mL/kg of WS (group I). The amounts of lidocaine and epinephrine in WS were also higher for group II, whereas postoperative pain scores, the number of patients receiving blood transfusions, and the lengths of hospitalization did not differ between the groups.

The super-wet technique (aspiration of 1 mL of fat per 1 mL of infiltrate) has been applied frequently in liposuction worldwide [1]. Epinephrine and lidocaine are always added to the WS because of their hemostatic and analgesic effects. One major advantage of the super-wet technique is that blood loss is quite low. However, the potential cardiovascular side effects of WSs and the amounts of epinephrine and lidocaine contained in the WSs, such as volume overload, local anesthetic toxicity, hypertension, arrhythmia, and tachycardia, continue to be investigated. In this study, we examined a WS and the medications it contains to understand the possible causes of these adverse outcomes. Although the most suitable candidates for liposuction are young adults with BMI <30 kg/m^2^ and few or no comorbidities, many patients undergoing liposuction are obese; thus, obesity-related pathophysiological changes after surgery must be considered. In this study, nearly 80% of the patients were overweight or obese. Therefore, we analyzed the data by dividing the patients into two groups according to their IBW-based WS amount.

In each patient undergoing surgery under anesthesia, the doses of all fluids and medications are calculated according to the patient’s weight in kilograms. However, the dose of WS is often calculated during liposuction according to the amount of fat aspirated. Epinephrine and lidocaine are administered in standard doses determined by each clinic for each liter of WS to be used. However, in obese and overweight patients, the volume of distribution and elimination function may affect the pharmacokinetic behavior of drugs [3,12]. Therefore, calculating a drug dose according to the patient’s current body weight or applying a standard dose regardless of the ideal weight may be unsafe.

Previous studies have reported low complication rates for liposuction procedures [13,14,15]. However, the presence of obesity and overweight (BMI of 25–30 kg/m^2^) has been identified as an independent predictor of all complications [16]. In the present study, the IBW values of the two groups were similar, yet the BMI of the patients in group II was significantly higher than in group I. In group II, 31.2% of patients had a BMI between 25 and 30 kg/m^2^, while 58.7% had a BMI above 30 kg/m^2^. This study aimed to identify the potential complications associated with this BMI difference.

The use of a greater volume of WS inevitably becomes unavoidable in the course of performing liposuction procedures on obese and overweight patients. However, the advantages and disadvantages of this increased use of WS and its components remain unclear. In particular, an ABW-based dosing regime in high-volume liposuction procedures for obese and overweight patients may result in excessive exposure to WS components (adrenaline and lidocaine). IBW is an alternative size descriptor used to determine drug dosage in many therapeutic applications in the perioperative period that is relatively easy to calculate [8,17].

Considering the potential variability in the pharmacokinetic properties of drugs in this patient group, the ratio of the WS and its components to IBW may prove a valuable guide in terms of postoperative complications. Furthermore, randomized controlled trials in which different WS and its components are formulated and compared according to IBW and ABW could be beneficial.

In this study, the total doses of lidocaine and epinephrine in the WS were significantly higher for patients in group II than for those in group I. No major postoperative pulmonary or cardiac complications occurred in either group, but in comparison with group I, group II exhibited significantly more postoperative nausea (*p* < 0.001) and vomiting (*p* = 0.041), had longer PACU stays (*p* = 0.006), and had higher rates of hypertension (*p* = 0.033) and hypothermia (*p* = 0.02).

In previous studies, epinephrine in doses ≤10 mg administered through the WS was used during liposuction without adverse events [1,18]. Klein and Jeske reported that the maximum safe doses of tumescent lidocaine were 28 mg/kg without liposuction and 45 mg/kg with liposuction in a tumescent infiltration procedure performed on volunteers [6]. However, although no adverse clinical events were reported in the patients in these studies, such high doses of epinephrine and lidocaine may not be appropriate routinely. Diluting epinephrine and lidocaine in a WS and applying them over time to an area with relatively low vascularity might delay systemic absorption and might ensure that plasma concentrations do not rise to toxic levels; however, heart rate, blood pressure, and pulmonary pressure increase in patients undergoing high-volume liposuction [19]. Because the amount of fat aspirate increases during high-volume liposuction, especially in obese and overweight patients, the amount of WS and thus the amounts of epinephrine and lidocaine also increase.

In this study, the ABW-based mean doses of epinephrine (2 mg for group I and 3.5 mg for group II) and lidocaine (30.5 mg/kg for group I and 42.6 mg/kg for group II) are within the safe dose limits reported in previous studies. The IBW values of the two groups were similar, but 90% of the patients in group II were obese or overweight. When calculated according to IBW, the mean epinephrine doses would be 0.0037 mg/kg for group I and 0.062 mg/kg for group II, and the mean lidocaine doses would be 36.7 mg/kg for group I and 61.4 mg/kg for group II. Although the doses calculated according to ABW are within the safe range reported in previous studies, the lidocaine dose calculated according to IBW exceeds the safe level. Nonetheless, it is more important to determine and apply the lowest dose that is effective and sufficient for the patient. The purpose of adding epinephrine, a vasoconstrictor, to the WS is to reduce blood loss, whereas lidocaine provides analgesia. Therefore, the minimum doses that can meet these purposes within safety limits should be used.

In this study, the amount of epinephrine in the WS was higher for group II than for group I, and the postoperative hypertension risk was 4.9-fold higher in group II as a result. Brown et al. showed that the plasma levels of epinephrine peaked between the first and fifth hours after WS administration, and approximately 25% was absorbed into the vascular system [18]. They reported that these plasma levels were approximately four times the resting epinephrine level (major physiological stress level). The amount of epinephrine absorbed into the systemic circulation may vary among individuals. In some patients, more of the drug than expected may pass into the plasma. Therefore, as the WS and the epinephrine content increase, the amount of epinephrine absorbed into the systemic circulation also increases. Increased epinephrine levels in plasma may lead to tachyarrhythmia, cardiac arrest, and myocardial infarction in patients with inadequate cardiac reserve [19]. Although preoperative hemoglobin levels were similar in both groups, postoperative blood transfusion rates were also similar. Thus, increasing the amount of epinephrine in patients who received >90 mL/kg of WS according to IBW did not reduce the need for postoperative blood transfusion and did increase the risk of postoperative hypertension.

Although more lidocaine was administered in group II, the postoperative pain scores were similar in both groups. Some studies have shown that lidocaine used as a short-acting local anesthetic reduces postoperative pain scores and intraoperative anesthetic consumption; however, its use in high-volume liposuction performed in patients under general anesthesia remains controversial because local anesthetic toxicity and cardiac and neurological adverse effects might occur [20,21,22]. Hatef et al. showed that despite increasing lidocaine doses, postoperative pain scores did not change and anesthetic consumption did not increase [23]. Similarly, in another study of individuals who did and did not receive lidocaine during liposuction on one half of their body, the pain scores did not differ. Clinicians must be aware that lidocaine doses, especially those calculated according to ABW or increased in parallel with the increase in the amount of fat aspirate in high-volume liposuctions, may reach toxic levels and may not provide an advantage in terms of postoperative pain [22].

All these data indicate that adverse events such as hypothermia, nausea, vomiting, and hypertension are more common in group II patients due to the administration of more epinephrine and longer operation time. Since plasma epinephrine levels were not measured, the contribution of hypothermia to postoperative hypertension should not be ruled out. However, due to the thermal balance-disrupting effect of epinephrine, deeper hypothermia may have been observed in group II patients who received more epinephrine [24].

This study had several limitations. First, all the high-volume liposuction procedures were performed by the same surgeon at our clinic; different WS protocols may yield different results. WS protocols involving different doses of epinephrine and lidocaine should be compared in randomized controlled studies. Comparisons of WS and their contents that are adjusted according to IBW and ABW may also provide valuable information for high-volume liposuction performed on patients with a BMI ≥30 kg/m^2^ Second, we monitored our patients throughout their hospitalization but not after hospital discharge; thus, only the short-term results are presented.

## 5. Conclusions

This retrospective analysis of patients who underwent high-volume liposuction revealed a 5.3-fold increased risk of nausea, a 4.9-fold increased risk of hypertension, and a 4.2-fold increased risk of hypothermia in those receiving >90 mL/kg of WS calculated according to IBW. During high-volume liposuction, especially in obese and overweight patients, the amounts of WS and its components (epinephrine and lidocaine) calculated according to IBW may reduce complications such as postoperative hypertension, nausea, vomiting, and hypothermia.

## Figures and Tables

**Table 1:** Patients’ characteristics.

Patients, n	192
Age, years	39 (31–47)
BMI, kg/m^2^, n (%)	29.5 (25.4–34.6)
<25	39 (20.3)
25–30	61 (31.8)
>30	92 (47.9)
IBW, kg	57 (52–60)
ASA score	1 (1–2)
Comorbidities, n (%)	
Hypothyroidism	40 (20.8)
Hypertension	9 (4.7)
Diabetes mellitus	7 (3.6)
COPD	3 (1.6)
Hemoglobin, gr/dL	
Preoperative	12.2 ± 1.2
Postoperative	9.6 ± 1.3
MAP, mmHg	
Before anesthesia induction	89 (80–100)
After anesthesia induction	75 (68–83)
<65 mmHg during operation	8 (4.2)
Administered wetting solution and components	
Wetting solution, L	6.3 (5.0–7.5)
Wetting solution/IBW, mL/kg	114 (90–138)
Lidocaine, g	3.1 (2.5–3.8)
Adrenaline, mg	3.3 (2.5–4.0)
Lipoaspirate volume, L	7.3 (6.0–8.5)
Duration of surgery, min	230 (200–265)
Administered IV fluid during the operation	
Total, mL	1850 (1500–2000)
Crystalloids, mL	1850 (1500–2000)
Colloids, mL	0 (0–0)
Body temperature at the end of the operation, °C	34.9 (34.2–35.3)
<34 °C	21 (10.9)
Urine output, mL	300 (200–500)
Duration of PACU, min	70 (55–84)
Postoperative complications, n (%)	
NRS > 5	43 (22.4)
Nausea	45 (23.4)
Vomiting	24 (12.5)
SpO2 < 90%	6 (3.1)
MAP > 100 mmHg	24 (12.5)
Red blood cell transfusion requirement	25 (13.0)
Length of the hospital stay, days	2 (1–2)

BMI, body mass index; IBW, ideal body weight; ASA, American Society of Anesthesiologist; MAP, mean arterial pressure; PACU, post-anesthetic care unit; NRS, numerical pain rating scale; IV, intravenous.

**Table 2:** Comparisons between WS/IBW groups.

	Group I (WS/IBW ≤ 90 mL/kg) (n = 54)	Group II (WS/IBW > 90 mL/kg) (n = 138)	*p*
Age, years	37 (30–43)	40 (31–49)	0.057
BMI, kg/m^2^	25.1 (23.6–28.6)	31.6 (27.3–35.5)	**<0.001**
<25	25 (46.3)	14 (10.1)	**<0.001**
25–30	18 (33.3)	43 (31.2)	
>30	11 (20.4)	81 (58.7)	**<0.001**
IBW, kg	57 (55–61)	56 (52–59)	0.108
ASA score	2 (1–2)	1 (1–2)	0.121
Comorbidities, n (%)			
Hypothyroidism	14 (25.9)	26 (18.8)	0.277
Hypertension	2 (3.7)	7 (5.1)	1.000
Diabetes mellitus	1 (1.9)	6 (4.3)	0.675
COPD	1 (1.9)	2 (1.4)	1.000
Hb, g/dL			
Preoperative	12.7 ± 1.1	12.8 ± 1.2	0.654
Postoperative	9.8 ± 1.2	9.7 ± 1.3	0.215
MAP, mmHg			
Before anesthesia induction	87 (78–95)	90 (81–101)	0.055
After anesthesia induction	74 (67–80)	75 (68–84)	0.115
<65 mmHg during operation, n (%)	2 (3.5)	4 (3.3)	1.000
Wetting solution, L	4.0 (3.5–5.0)	7.0 (6.0–8.0)	**<0.001**
Lidocaine, g	2.0 (1.8–2.5)	3.5 (3.5–4.3)	**<0.001**
Lidocaine, mg/kg (ABW)	30.5 (23.7–33.4)	42.6 (36.4–47.3)	**<0.001**
Lidocaine, mg/kg (IBW)	36.7 (32.1–42.6)	61.4 (55.0–74.7)	**<0.001**
Epinephrine, mg	2.0 (1.8–2.5)	3.5 (3.0–4.0)	**<0.001**
Epinephrine, mg/kg (ABW)	0.031 (0.024–0.034)	0.043 (0.036–0.047)	**<0.001**
Epinephrine, mg/kg (IBW)	0.037 (0.032–0.044)	0.062 (0.055–0.075)	**<0.001**
Lipoaspirate volume, L	5.0 (4.5–6.0)	8.0 (7.0–9.0)	**<0.001**
Liposuction areas, n (%)			**<0.001**
Two area	45 (83.3)	21 (15.2)	
Three areas	8 (14.8)	65 (47.1)	
Five areas	1 (1.9)	52 (37.7)	
Duration of surgery, min	200 (184–226)	240 (214–280)	**<0.001**
Body temperature at the end of the operation, °C	35.0 (34.5–35.5)	34.7 (34.1–35.2)	**0.002**
<34 °C, n (%)	2 (3.7)	19 (13.8)	**0.033**
Urine output, mL	300 (200–400)	325 (200–500)	0.178
Duration of PACU, min	60 (50–71)	75 (55–90)	**0.006**
Postoperative complications, n (%)			
NRS > 5	10 (18.5)	33 (23.9)	0.420
Nausea	4 (7.4)	41 (29.7)	**<0.001**
Vomiting	3 (5.6)	21 (15.2)	**0.041**
SpO2 < 90%	1 (1.9)	5 (3.6)	1.000
MAP > 105 mmHg	2 (3.7)	22 (15.9)	**0.021**
Red blood cell transfusion requirement	7 (13.0)	18 (13.0)	1.000
Length of hospital stay, days	2 (1–2)	2 (1–2)	0.095

WS, wetting solution; BMI, body mass index; IBW, ideal body weight; ABW, actual body weight; ASA, American Society of Anesthesiologists; Hb, hemoglobin; MAP, mean arterial pressure; PACU, post-anesthetic care unit; NRS, numerical pain rating scale.

**Table 3:** Relative risks of nausea, hypothermia, and hypertension in patients with WS/IBW > 90 mL/kg.

	RR (CI 95%)	*p*
Nausea	5.3 (1.8–15.6)	**0.003**
Hypertension (>105 mmHg)	4.9 (1.2–19.8)	**0.027**
Hypothermia (<34 °C)	4.2 (1.1–18.5)	**0.045**

WS, wetting solution; IBW, ideal body weight.

## Data Availability

The datasets for this study are available from the corresponding author upon reasonable request.
